# In Memoriam

**Published:** 1903-07

**Authors:** 


In Memoriam.—Dr. I. N. Love—dear, delightful Love, is dead! A star of the first magnitude is blotted out of the firmament; the profession of medicine, the teachers' guild, the fraternity of medical editors, are prematurely robbed of one of their brightest, best, most valued, most loved members. Oh, the pity of it! That he should have been so suddenly, so unexpectedly, cut off in the prime of his mature manhood, in the zenith of his fame, at ebb-tide of his personal popularity! We mourn his loss, and will not be comforted. Dr. Love was the most magnetic, the most lovable of men. His personal influence was something remarkable. He drew men to him irresistibly, and his friends all over the world are "like the leaves of the forest when summer is green." "None knew him but to love him, none named him but to praise." A long farewell—dear, genial, beloved I. N. Love!

Dr. Love was 55 years of age. He was a graduate of the Medical Department of Washington University, St. Louis, of the class of 1872, a protege and student of the celebrated Professor Hodgson. He had been President of the American Medical Editors' Association; member for many years of the Board of Trustees of the American Medical Association; member of the Executive Committee of the Mississippi Valley Medical Association; first vice-president of the American Medical Association; member of the Board of Trustees of the First Pan-American Medical Congress; Professor of Diseases of Children, Clinical Medicine and Hygiene in Marion-Sims Medical College; and at the time of his death was a successful practitioner in New York City and teacher in one of the post-graduate schools and hospitals. He founded *Love's Medical Mirror* in St. Louis fourteen years ago, made a beautiful and brilliant success of it, and to the date of his lamented death it delighted its thousands of readers by its wholesome, cheering optimism. Dr. Love was returning from Paris, where he had been as physician to a wealthy lady, and died suddenly on board of the steamer "Aurania" just before she entered the harbor at New York. Mrs. Love is a Texas woman, of Marshall, Texas. The last thing Dr. Love did before he was stricken down was to make a speech. He was famous for delightful extemporaneous speeches.

